# Establishing a Sickle Cell Disease Registry in Africa: Experience From the Sickle Pan-African Research Consortium, Kumasi-Ghana

**DOI:** 10.3389/fgene.2022.802355

**Published:** 2022-02-24

**Authors:** Vivian Paintsil, Evans Xorse Amuzu, Isaac Nyanor, Emmanuel Asafo-Adjei, Abdul Razak Mohammed, Suraj Abubakar Yawnumah, Yaa Gyamfua Oppong-Mensah, Samuel Blay Nguah, Paul Obeng, Elliot Eli Dogbe, Mario Jonas, Victoria Nembaware, Gaston Mazandu, Kwaku Ohene-Frempong, Ambroise Wonkam, Julie Makani, Daniel Ansong, Alex Osei-Akoto, the Sickle Cell Disease in Sub-Saharan Africa Consortium

**Affiliations:** ^1^ Directorate of Child Health-Komfo Anokye Teaching Hospital, Kumasi, Ghana; ^2^ Department of Child Health-Kwame Nkrumah University of Science and Technology, School of Medicine and Dentistry, Kumasi, Ghana; ^3^ Department of Pathology, Division of Human Genetics, Faculty of Health Sciences, University of Cape Town, Cape Town, South Africa; ^4^ Sickle Cell Foundation of Ghana, Kumasi, Ghana; ^5^ SPARCo, Sickle Cell Programme, Muhimbili University of Health and Allied Sciences, Dar Es Salam, Tanzania

**Keywords:** sickle cell disease, registry, SPARCo, SickleInAfrica, Kumasi-Ghana

## Abstract

Sickle cell disease (SCD) is the most common clinically significant hemoglobinopathy, characterized by painful episodes, anemia, high risk of infection, and other acute and chronic complications. In Africa, where the disease is most prevalent, large longitudinal data on patients and their outcomes are lacking. This article describes the experiences of the Kumasi Center for SCD at the Komfo Anokye Teaching Hospital (KCSCD-KATH), a Sickle Pan-African Research Consortium (SPARCO) site and a SickleInAfrica Consortium member, in establishing a SCD registry for the evaluation of the outcomes of patients. It also provides a report of a preliminary analysis of the data. The process of developing the registry database involved comprehensive review of the center’s SCD patient medical records, incorporating data elements developed by the SickleInAfrica Consortium and obtaining ethical clearance from the local Institutional Review Board. From December 2017 to March 2020, 3,148 SCD patients were enrolled into the SCD registry. Enrollment was during the SCD outpatient clinic visits or through home visits. A significant proportion of the patients was from the newborn screening cohort (50.3%) and was males (52.9%). SCD-SS, SCD-SC, and Sβ ^+^thalassemia were seen in 67.2, 32.5, and 0.3% patients, respectively. The majority of the patients were in a steady state at enrollment; however, some were enrolled after discharge for an acute illness admission. The top two clinical diagnoses for SCD-SS patients were sickle cell painful events and acute anemia secondary to hyperhemolysis with incidence rates of 141.86 per 10,000 person months of observation (PMO) and 32.74 per 10,000 PMO, respectively. In SCD-SC patients, the top two diagnoses were sickle cell painful events and avascular necrosis with incidence rates of 203.09 per 10,000 PMO and 21.19 per 10,000 PMO, respectively. The SPARCO Kumasi site has developed skills and infrastructure to design, manage, and analyze data in the SCD registry. The newborn screening program and alternative recruitment methods such as radio announcement and home visits for defaulting patients were the key steps taken in enrolling patients into the registry. The registry will provide longitudinal data that will help improve knowledge of SCD in Ghana and Africa through research.

## Introduction

Sickle cell disease (SCD) is one of the most common inherited blood diseases, which affect red blood cells (RBCs) in humans. The disease is characterized by painful episodes, anemia, high risk of infection, and other acute and chronic complications (Williams et al., 2009; Grosse et al., 2011; Scott et al., 2011). Worldwide estimates indicate that 20–25 million people are affected by SCD with approximately 60% of those affected, living in sub-Saharan Africa (Aliyu et al., 2008). SCD is most prevalent in sub-Saharan Africa (Munung et al., 2019) with an estimated number of 240,000 patients born with SCD-SS each year (Weatherall, 2011). Ghana accounts for about 15,000 (6.25%) of that number (Ohene-Frempong et al., 2008). The global number of newborns with SCD is estimated to increase to about 400,000 by the year 2050 with 85% expected to be born in sub-Saharan Africa (Piel et al., 2013a). Implementation of early diagnosis of SCD in newborns and a comprehensive management plan including penicillin prophylaxis, vaccination, disease-modifying drugs, screening for and prevention of complications, supported with health maintenance have been shown to significantly reduce mortality and prolong the life of patients (Piel et al., 2013a; Kwarteng-Siaw et al., 2017). In Africa, there are limitations in standards of care for management of SCD, skills development for health care professionals in SCD, and comprehensive databases of patients for monitoring clinical care (Diallo and Tchernia, 2002; Wonkam and Makani, 2019; Oron et al., 2020; RFA-HL-17–006, 2021a).

The Kumasi Center for SCD at the Komfo Anokye Teaching Hospital (KCSCD-KATH) was inaugurated in 1992 as a comprehensive SCD management center in preparation for the start in 1993 of the NHLBI-funded pilot research project entitled, “Newborn Screening for Sickle Cell Disease in Ghana”, the first public health-based newborn screening project for SCD in Africa.

In September 2015, the National Heart, Lung, and Blood Institute (NHLBI) of the National Institutes of Health (NIH) of the United States of America issued a “request for application” for establishment of a “Sickle Cell Disease in Sub-Saharan Africa: Collaborative Consortium” (RFA-HL-17–006, 2021b) and a related request for a “Sickle Cell Disease in Sub-Saharan Africa: Data Coordinating Center” (RFA-HL-17–006, 2021a). A multinational collaboration among Muhimbili University of Health and Allied Sciences (MUHAS), Dar Es Salaam, Tanzania, as the hub, and two additional sites, the University of Abuja, Abuja, Nigeria, and the Kwame Nkrumah University of Science and Technology (KNUST), Kumasi, Ghana, applied for and were awarded the consortium grant. The University of Cape Town, Cape Town, South Africa, was the successful applicant for the data coordinating center. The consortium was named “Sickle Pan-African Research Consortium” (SPARCO) and the data coordinating center was named Sickle Africa Data Coordinating Center (SADaCC) and later together labeled SickleInAfrica (Makani et al., 2020) (https://www.sickleinafrica.org).

The main objectives of this consortium are:

• Formation of a centralized, electronic, patient-consented, sickle hemoglobinopathy database that will facilitate registration and follow-up of SCD patients and serve as the backbone for future SCD in sub-Saharan Africa research network operations.
• Creation of shared database elements as well as harmonized SCD phenotype definitions and ontologies.
• Integration of collaborative consortium activities with those of the Data Coordinating Center, National Heart, Lung, and Blood Institute (NHLBI), Steering Committee, Observational Monitoring Board, and any existing training programs.
• Development of SCD standards of care appropriate to regional resource availability and clinical needs.
• Organization of research and clinical skills development activities.
• Planning for future SCD cohort studies, implementation of preventive/therapeutic practices, and the inclusion of new African sites to constitute the SCD in the SSA research network.
• Accomplishment of collaborative consortium activities over 4 years, with a 2-year development phase, a 1-year pilot/planning phase, and a 1-year implementation phase.

Registries collect longitudinal data on patients and help in a better understanding of their experiences and clinical outcomes and assist in improving their health and well-being (Heeney et al., 2011). Registries are inherently observational which help them to describe the status quo and guide discussion on research and any possible interventions. Registries have been found to provide information that helps in identifying gaps and informing policy and strategies.

One of the most ambitious registries is the SECURE-SCD registry. In the midst of the COVID-19 pandemic, the SECURE-SCD registry has been developed to gather information on SCD patients, who develop COVID-19 worldwide. This registry gathers reported data from health providers (Panepinto et al., 2020).

In the USA, several registries exist including Cooperative Study of Sickle Cell Disease (CSSCD) (Farber et al., 1985), Comprehensive Sickle Cell Centers Clinical Trial Consortium (de-Graft Aikins et al., 2010), Children’s Hospital of Pittsburgh Sickle Cell Research registry (Hillery, 2021), the European Hemoglobinopathy registry (European Haemoglobinopathy Registry » Sickle Cell Society, 2021), the Sickle Cell Disease Implementation Consortium (SCDIC) program (DiMartino et al., 2018; Glassberg et al., 2020), and Sickle Cell Clinical Research and Intervention Program (Hankins et al., 2018).

The Sickle Cell Disease Implementation Consortium (SCDIC) program was also set up in 2016 in the United States of America (USA) to support implementation research in SCD. The 6-year registry project targets 2,400 patients specifically of ages 15–45 across eight centers and captures standardized clinical measures and also patient experiences (DiMartino et al., 2018; Glassberg et al., 2020).

The SCCRIP started in 2014 as a 30-year program aimed at enrolling 10,000 participants across all age groups in five sites and has a pharmacokinetics sub-study to investigate the responses to hydroxyurea (Hankins et al., 2018).

Though SCD is more prevalent in sub-Saharan Africa, there is little data published from registries that gather longitudinal data on patients. Registries require a lot of resources to set up, recruit patients, and maintain over time as longitudinal data are required.

In Africa, prior to the SPARCO initiative, several efforts have been made in establishing various kinds of SCD registries including the Sickle Cell Disorder Registry Nigeria (SCDRN) (Sickle Cell Foundation Nigeria, 2021), Muhimbili University of Health and Allied Sciences, Tanzania (Makani et al., 2018), Sickle Cell Disease Genomics Network of Africa (SickleGenAfrica), Makerere University, Kenya, University of Cape Town, South Africa, University Teaching Hospitals, Children’s Hospital, Zambia, University of Rwanda, School of Medicine and Pharmacy, University of Zimbabwe College of Health Sciences, DELGEME, Mali, and University of Abuja, Nigeria.

The Sickle Cell Disorder Registry Nigeria is a collaboration between the Sickle Cell Foundation Nigeria and PointCareHealth Initiative aimed at collecting patient clinical information and patient-reported information across Nigeria, to support management of the patients as well as stimulate research into interventions (Sickle Cell Foundation Nigeria, 2021).

The KCSCD-KATH since its inception developed a clinical database using FileMaker Pro to manage outpatient and inpatient encounters of its cohort of newborn-screened patients. The database has helped in tracking newborns from screening through to their enrollment and management in the clinic.

Registries are varied based on their purpose, target population, duration of enrollment, and the numbers of recruiting sites. This has raised calls for the standardization of the collection of data in these registries to provide more valuable information from large patient populations across countries (McCormick et al., 2021).

SADaCC and SPARCO Hub are building a robust electronic platform to support the activities of SPARCO. SADaCC and SPARCO Hub are responsible for the creation and maintenance of this centralized, electronic sickle hemoglobinopathy database and the development of data management, bioinformatics, and biostatistical skills across the consortium sites (Makani et al., 2020). This database is being designed to collate deidentified data from the consortium sites while sites manage their comprehensive registries. As a precursor to the centralized database, an appropriate SCD registry has been implemented to monitor and document the patient recruitment and follow-ups in Ghana. The registry is generating data for the evaluation of the outcomes of patients and the quality of care provided and hence assists in developing better standards of care for patients. The availability of the registry can enhance research, education, policy, and public health programs that will improve patient outcomes (de Groot et al., 2017).

This report describes the efforts of SPARCO-Kumasi in establishing a SCD patient registry and provides a report of a preliminary analysis of the data generated so far.

## Methods

### Study Site

The study was conducted at the Kumasi Center for Sickle Cell Disease, Komfo Anokye Teaching Hospital (KCSCD-KATH), affiliated with KNUST. KATH is located in Kumasi metropolis, the regional capital of the Ashanti Region and the most populous metropolitan area with 3.348 million inhabitants (Macrotrends. Accra, 2021). The hospital is a tertiary hospital with a bed capacity of 1,200 which serves as a major referral center for 12 out of 16 regions of Ghana (About Us | Komfo Anokye Teaching Hospital, 2021).

Since the first babies were tested in February 1995 at KATH, the hospital has screened more babies for SCD than any other site in Africa. Approximately 8,000 presumptive SCD patients have been identified by the newborn screening program (Ohene-Frempong, 2017) and approximately 80% of these patients were enrolled in the SCD clinic. The KCSCD-KATH offers predominantly pediatric services. Newborn babies with SCD are enrolled at the KCSCD by 2–3 months of age and started on twice daily penicillin prophylaxis, infant series of pneumococcal conjugate vaccine, once daily folic acid, and parental or caretaker education about SCD management in young children. Patients up to 3 years of age are scheduled to be reviewed every 2 months and those above 3 years, every 3 months *as* specified in the standard operating procedure of the center. Routine health maintenance evaluations include blood tests, complete blood counts with reticulocyte count, metabolic panel to assess kidney and liver functions, and eye screening for adolescents. Transcranial Doppler ultrasonography is available for patients but on a user-fee basis. Hydroxyurea therapy, previously offered to patients who could afford, is currently being offered for free to all patients under a public–private Partnership between Government of Ghana and Novartis AG (Novartis, 2019; Ministry of Health G, 2020). Since January 2019, KATH has established an electronic health record system for both outpatient and inpatient encounters.

### Database Design

The SPARCO-Kumasi site aimed to enroll 3,000 SCD patients over a 4-year period (2017–2020) and entered their basic demographic and clinical details into an electronic database system. The Research Electronic Data Capture (REDCap) platform was chosen for reasons including its flexibility, ease of design, interoperability, security features, and the ability to switch between web-based and mobile data collection platform in cases where internet was unavailable (Harris et al., 2009; Harris et al., 2019).

Basic data elements from the FileMaker Pro database of the KCSCD-KATH served as a primary source to build the subsequent registry. Subsequently, the Kumasi site initiated steps to design a locally relevant database pending the joint effort at developing a centralized hemoglobinopathy database for SPARCO hosted by SADaCC.

The first step in the process was a review of the SCD-specific patients’ chart that was in use for documenting patient clinical encounters at the KCSCD-KATH. The review also took into consideration the clinic visit documentation of the Sickle Cell Program of *the* Muhimbili National Hospital in Dar Es Salaam, Tanzania. A revised chart was then developed incorporating all the necessary changes. The document was then submitted to the Biostatistics Unit of KATH to be incorporated into the hospital’s electronic health management system.

The final version of the chart was used in designing the REDCap database. The data elements received from SickleInAfrica were also incorporated to modify the database as required to allow for future data harmonization with the SPARCO/SADaCC registry database. Patient identifiers were appropriately indicated in the REDCap database. Default values were also inserted as appropriate values to ease data encoding. Dummy records were entered to assess the reliability, validity, and precision of the database in the collection of patient information.

### Patient Enrollment and Data Collection

All SCD patients reporting for clinical care at the KCSCD-KATH were eligible for enrollment.

Study procedures were explained to patients and/or their caregivers, and those who agreed to participate were asked to sign a written informed consent form to show their approval before they were enrolled into the registry. Informed consent was obtained from caregivers of patients (if the patient was below 18 years) or from patients themselves (if patients were 18 years and over). Assent was obtained from patients aged 7–17 years in addition to caregivers’ written informed consent.

Information on hemoglobin phenotype, date of birth/age at enrollment, sex, religion, residence, penicillin V, folic acid, and hydroxyurea usage were collected through interview with the patient/caregiver and review of medical records of the patients. The diagnosis pathway was recorded as either via newborn screening or non-newborn screening. Patients whose hemoglobin phenotypes were diagnosed by the newborn screening were tested using the iso-electric focusing method. Those who were diagnosed beyond the newborn age were tested using the Hb electrophoresis method. S/beta-plus thalassemia would show Hb S > Hb A. Information on the hemoglobin phenotypes was obtained from the patient records. The patients were not tested routinely for alpha-thalassemia and so this was not captured. No genetic testing was performed on patients who were enrolled. Follow-up clinical encounters at each clinic visit were also captured. Patients’ diagnosis was classified according to the SCD ontology definitions (Sickle Cell Disease Ontology, 2020), and the following common to the cohort was defined subsequently as in Table 1.

**TABLE 1:** Definition of SCD terms.

Name	Definition
SCD-SS	A variant of SCD due to homozygosity of the *E6V* mutation, the amino acid substitution of valine for glutamic acid in the sixth position of the beta-globin chain, resulting in the production of hemoglobin S from both alleles
SCD-S beta-zero thalassemia	A form of sickle cell thalassemia characterized by the absence of hemoglobin A. Patients usually have severe anemia identical to that seen in sickle cell disease
SCD-S beta-plus thalassemia	A mild form of sickle cell thalassemia characterized by the presence of hemoglobin S and a reduced amount of hemoglobin A in the red blood cells. It is characterized by the presence of small red blood cells and mild anemia
SCD-SC	A type of sickle cell disease characterized by the presence of both hemoglobin S and hemoglobin C. It is similar to, but less severe than SCD-SS
Steady state	That period when the patient with sickle cell disease is not experiencing infections, pain, or other acute disease complications
Sickle cell painful event	Pain lasting at least 2 hours that requires an unscheduled emergency room visit or hospitalization or that disrupts daily activities
Acute chest syndrome (ACS)	A lung disease that involves a vaso-occlusive crisis of the pulmonary vasculature seen in patients with sickle cell disease
Nonspecific acute lower respiratory tract episodes	Includes acute respiratory episodes with lower respiratory tract signs that do not meet the criteria for other diagnoses. May include episodes which would have been diagnosed as ACS where radiographic facilities are available
Acute anemia	A “generic” term for a sudden drop (often defined as 20% or more) in the Hb level beyond the baseline and divided into three common pathophysiologic types: “acute splenic sequestration”, “transient erythroid aplasia” (most commonly due to parvovirus ^19^B infection), and “acute hemolysis” of various causes
Hyperhemolysis	Significant change in the blood picture characterized by a precipitous fall in the hemoglobin level associated with jaundice, marked reticulocytosis and polychromasia on the blood smear, and increased unconjugated hyperbilirubinemia and increased urobilinogen content in urine above the steady state level for each individual patient
Malaria	An individual with malaria-related symptoms (fever axillary temperature ≥ 37.5°C, chills, severe malaise, headache, or vomiting) at the time of examination or 1–2 days prior to the examination in the presence of a *Plasmodium*-positive blood smear or a positive malaria rapid diagnostic test
Cerebrovascular accident (CVA)	Characterized by sudden loss of the neurological function due to brain ischemia or intracranial hemorrhages. Presents with sudden onset of weakness, aphasia, and sometimes seizures or coma and results in adverse motor and cognitive sequelae
Avascular necrosis (AVN)	Also known as aseptic necrosis, osteonecrosis, or ischemic necrosis is bone death due to compromised blood supply. The hip joint is the most common site of AVN
Dactylitis	A severe acute inflammatory response affecting the hands and feet of individuals with sickle cell disease. It is caused by vaso-occlusive episodes leading to ischemia and finally infarction of the distal portions of the extremities. Clinical signs of pain, swelling, and tenderness of digits usually begin in early childhood and may be the initial manifestations of sickle cell anemia
Sepsis	The body’s severe inflammatory response to infection and mostly presents with fever. Infections that lead to sepsis most often start in the lung, urinary tract, skin, or gastrointestinal tract
Priapism	A sustained unwanted painful erection lasting 4 or more hours and not associated with sexual activity
Osteomyelitis	An inflammatory process accompanied by bone destruction and caused by an infecting microorganism

### Alternative Enrollment Methods

Alternative methods were initiated in January 2019 to enroll patients who had defaulted in clinic visits. A patient was defined to have defaulted if the last clinic attendance was more than a year. The first method was an invitation by radio announcements for a mass enrollment drive over two days in January 2019. Those who responded and presented to the SCD outpatient clinic were registered. The second method involved home visits using the KCSCD existing clinical database. Information on patients who had defaulted in clinic attendance was extracted. Patients were initially contacted on phone, given an appointment, and invited to attend the clinic by trained research assistants. A second contact was made if they failed to honor the clinic appointment, and then a scheduled home visit was made. Same enrollment procedures as described earlier were followed during the home visit. The visit also offered the opportunity to encourage and reschedule the patients to return to the clinic. The home visits started in March 2019.

### Data Management

Data quality checks were carried out weekly, and queries were resolved. Data on the server were backed up unto an external hard disk system aside the daily automatic backups of the server. Quarterly automatic backups from the online server were saved online and on a separate external hard disk. Deidentified data were also securely sent to the SPARCO Hub and SADaCC as a form of backup using REDCap.Data were exported from the REDCap system in Stata format and imported into Stata 14.0 (StataCorp 4905 Lakeway Drive Station Texas 77845, United States) for analysis. Basic descriptive analysis was carried out on the data and was presented as appropriate frequency tables and figures.

We estimated the incidence rate (IR) as a rate at which new clinical events (diagnosis) occurred after enrollment of the patients. The total period of observation (person-time at risk) was calculated in months for each SCD genotype (i.e., SCD-SS, SCD-SC, and SCD S beta-plus thalassemia). The IR was evaluated per 10, 000 person-months with 95% confidence interval (C.I.) due to the short observance period. The IRs with their 95% C.I. were generated using an online C.I. calculator for the single incidence rate from the Chinese University of Hong Kong (C.I. for Single Rate, 2022).

### Ethical, Legal, and Social Issues

Ethical approval was obtained from the Committee on Human Research Publications and Ethics, a joint committee of the School of Medical Sciences of KNUST and KATH. In consonance with agreed consortium principles, some key ethical, legal, and social issue elements that had been identified and discussed were incorporated into the informed consent and assent forms (Munung et al., 2019). Among these were the importance of informed consent and the type of informed consent obtained, the benefits of the study for the study population, and research priorities setting. These were found to be important to guide the use of the registry for research both locally and for the consortium.

## Results

From December 2017 to the end of March 2021, 3,148 SCD patients were enrolled into the SPARCO SCD Registry database. This constitutes 104.9% of the targeted 3,000.

Of the total number of patients enrolled, 1,582 (50.3%) were diagnosed from the newborn screening program. Most patients, i.e., 2,016 (64%) of them were enrolled when they reported to the outpatient clinic or upon discharge after an acute illness admission, while the rest 1,132 (36%) patients were enrolled through radio advertisement and home visits (alternative methods of enrollment).

More male patients were enrolled into the database (n = 1,665, 52.9%). Patients with the SCD-SS phenotype formed 67.2*%* of those recruited*.* Patients aged 5–9 years were the most common (29.1%) age group recruited, whilst those less than 5 years of age formed 27.6% of those recruited. Patients who were 15 years and older formed 17.3% of those recruited (Table 2).

**TABLE 2:** Demographic characteristics of patients.

Characteristic	Frequency	Percentage
Sex		
Female	1,483	47.1
Male	1,665	52.9
Age category		
0—4 + years	869	27.6
5–9+ years	915	29.1
10–14 + years	821	26.1
15–17 + years	258	8.2
≥18 years	285	9.1
SCD phenotype		
SCD-SS	2,116	67.2
SCD-SC	1,023	32.5
Sβ+Thal	9	0.3

**FIGURE 1:**
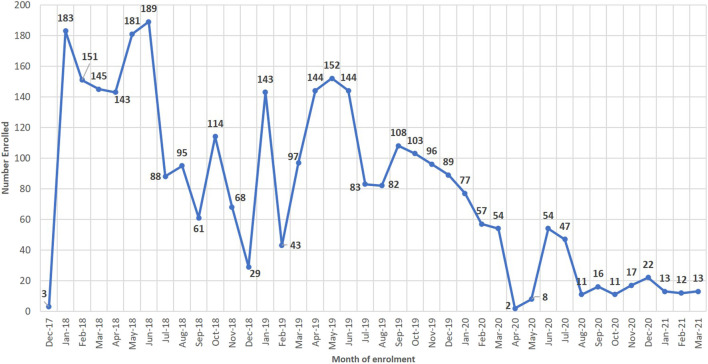
Enrolment trends into the registry.

The highest number enrolled in a month was in June 2018 (189) and the lowest in December 2017 (3), when the registration was started. Peaks were also realized when alternative methods of enrollment were implemented in January and March 2019. (Figure 1).

The enrolled patients had 24,116.22 months of 18,328.14 PMO while the 1,023 SCD-SC patients contributed 5,662.55 PMO. The average period of observation was 8.7 months for SCD-SS patients and 5.5 months for SCD-SC patients groups.

In this period, among the SCD-SS patients, the top two diagnoses were sickle cell painful events with an incidence rate (IR) of 141.86 per 10,000 PMO and severe anemia secondary to hyperhemolysis (IR = 32.74 per 10,000 PMO). In SCD-SC patients, the top two diagnoses were sickle cell painful events (IR = 203.09 per 10,000 PMO) and avascular necrosis (IR = 21.19 per 10,000 PMO). The incidence rate per 10,000 PMO of cerebrovascular accident (CVA) was 12.55 in SCD-SS patients and 3.53 in SCD-SC patients and that of acute chest syndrome was 7.64 in SCD-SS and 5.30 in SCD-SC patients (Table 3).

**TABLE 3:** SCD-related diagnosis.

Diagnosis	Incidence rate per 10,000 person months (95% CI)		
	SCD-SS (total person months = 18, 328.14)	SCD-SC (total person months = 5, 662.55)	SCD S beta-plus thalassemia (total person months = 125.52)
Sickle cell painful event	141.86 (125.73, 160.06)	203.09 (169.48, 243.36)	159.34 (40.29, 630.08)
Severe anemia secondary to hyperhemolysis	32.74 (25.43, 42.15)	14.23 (7.07, 28.24)	0
Acute malaria	17.46 (12.35, 24.68)	12.36 (5.90, 25.92)	0
Cerebrovascular accident (CVA)	12.55 (8.34, 18.88)	3.53 (0.88, 14.12)	0
Acute chest syndrome (ACS)	7.64 (4.53, 12.90)	5.30 (1.71, 16.42)	0
Avascular necrosis (AVN)	7.09 (4.12, 12.21)	21.19 (12.04, 37.29)	0
Dactylitis	5.46 (2.94, 10.14)	3.53 (0.88, 14.12)	79.67 (11.31, 561.17)
Sepsis	3.82 (1.82, 8.01)	7.06 (2.65, 18.82)	0
Priapism	2.73 (1.14, 6.56)	1.77 (0.25, 12.54)	0
Osteomyelitis	2.18 (0.82, 5.81)	1.77 (0.25, 12.54)	0

SCD-SS, sickle cell disease SS; SCD–SC, sickle cell disease SC.

## Discussion

The SPARCO-Kumasi, Ghana, has developed a comprehensive registry (Supplementary Table S1) capable of collecting and managing information on the clinic encounters of patients. The registry is being harmonized in collaboration with the SADaCC to ensure it is universally acceptable and accurately captures available data based on the SCD ontology. The standard operating procedures have been developed in collaboration with SPARCO and SADaCC to ensure uniformity in data collection and data quality and also compliance with the FAIR (Findable, Accessible, Interoperable, and Reusable for humans and computers) principles. SPARCO and SADaCC have also provided and supported training in the management of the database and analysis of data. The processes undertaken fairly meet the recommendations for disease registries (de Groot et al., 2017; Kodra et al., 2018).

The results reveal a significant proportion of patients enrolled in the SPARCO registry was identified through the newborn screening program in Ghana. Several initiatives for newborn screening for SCD have been instituted in Africa, but no country has successfully implemented a universal national newborn screening for the SCD program for early diagnosis of SCD (Hsu et al., 2018; Nkya et al., 2019). The most extensive and longest running program has been implemented in Ghana at our center spanning 25 years, and over 9,000 SCD patients have been identified through this method and enrolled for care at our center (Ohene-Frempong et al., 2008; Hsu et al., 2018; Nkya et al., 2019). In the most developed countries, however, most, if not all patients, are identified through newborn testing (Telfer et al., 2007), whereas in countries such as Africa most SCD patients are identified following an acute illness.

In our study, we saw male dominance as also alluded to in registries in Brazil and England (Telfer et al., 2007; Fernandes et al., 2015), but different from registries in Nigeria (Isa et al., 2020) and Accra-Ghana (Asare et al., 2018) in which there was female dominance. This could have been due to the sociocultural practices and health-seeking behaviors in the setting and not necessarily due to genetics and actual population prevalence.

The study reported a large proportion of patients below and within the adolescent age group. The significant proportion of children under 5 years highlights the positive impact of the newborn screening program in which patients identified are enrolled early into the SCD clinic for comprehensive care. The dominance of adolescents reinforces the need to urgently formulate a transition plan including appropriate education of adolescents (Kanter and Kruse-Jarres, 2013; Kwarteng-Siaw et al., 2017; Kwarteng-Siaw et al., 2019) and parents and strengthening adolescent and adult clinics to provide the continuum of care required. The lower number of patients aged over 18 years is attributable to the fact that the enrollment was initially focused on the pediatric sickle cell clinic. The scope of enrollment has now been expanded to cover the adult sickle cell clinic.

The dominance of the SCD-SS phenotype in the population matches several reports (Bardakdjian-Michau et al., 2009; de Castro Lobo et al., 2014; Fernandes et al., 2015; Asare et al., 2018; Isa et al., 2020). It is known that the prevalence of Hb S is high in Africa (Thachil et al., 2013) and specifically in countries below the equator in sub-Saharan Africa (Grosse et al., 2011). In our registry, the proportion of SCD-SS is higher by about 10% compared with data from the newborn screening program in Kumasi reported in 2005 (Ohene-Frempong et al., 2008) and subsequently in unpublished data in 2020. This is also comparable to the adolescent and adult population in Accra reported in 2019 (Asare et al., 2018). The increase in the proportion of SCD-SS in the 2020 report as compared to the 2005 report could be as a result of the inclusion of patients not screened during the newborn period in the current report. They are possibly the ones who will seek care because of the more severe course of the disease and therefore having a higher chance of being enrolled as compared to those with SCD-SC. In our population, there is a unique significant proportion of SCD-SC phenotypes similar to that of the Korle-Bu Teaching Hospital in Accra also in Ghana (Asare et al., 2018) but far higher than that found in Nigeria (Isa et al., 2020). The Hb C is known to be unique to West Africa and more commonly in Mali, Burkina Faso, and Ghana (Grosse et al., 2011; Piel et al., 2013b). In contrast to the situation of Ghana, Burkina Faso has a reverse of the dominance seen in the SCD-SS group in a similar ratio (Kafando et al., 2005).The high level of SCD-SC in West Africa possibly ameliorates the overall severity of SCD in the region. The lower proportion of SCD-SC in our registry compared with the newborn screening SCD-SC population (Ohene-Frempong, 2017) could be due to the milder clinical course, coupled with the health-seeking behavior of affected persons.

Enrollment had fluctuations reflecting patient adherence to clinic visits and the weather pattern. The rainy season in Ghana is between April and July. The wettest month is June, with June 2018 recording one of the highest rainfalls that resulted in flooding in Kumasi (Kumasi, 2020). The rainfall, cold, and humid conditions in this season predispose SCD patients to pain episodes and could have accounted for the peaks between April and June. April also coincides with the vacation periods for the most basic and secondary (senior high) schools, while June also coincides with the vacation periods for tertiary institutions. During these times, most of the SCD cohorts who are students are available for clinics. The other peaks were due to the initiation of enrollment methods: January 2018—start of clinic enrollments, January 2019—mass enrollment, and March 2019—home visits. The alternative methods were rolled out to actively seek the patients who were not adhering to their routine clinic visit schedules. There was a gradual decrease from October to December over the years. In December, there are longer public holidays, and most families spend the Christmas festive season away from their usual residences, a phenomenon also reported by Asare et al. (Asare et al., 2018). Inclusion into the study declined in the last year of the study. During the last year, there was the outbreak of COVID-19 which hampered attendance at the clinic. The alternative methods of recruitment using the home visits were also stopped for staff and patient safety. All these could have led to the reduction in the numbers that was recruited toward the end of the study.

The sickle cell painful event was the most reported event (Powars et al., 2005; Telfer et al., 2007; Kanter and Kruse-Jarres, 2013; Shah et al., 2019) at the outpatient clinic. The incidence rates in these studies were diverse which can be attributed to the designs and the populations of focus. Compared with a study in East London published in 2007 (Telfer et al., 2007) which had a similar design, the painful events incidence rate in our cohort was lower for SCD-SS patients but higher for SCD-SC patients. This is contrary to what is known in the literature. This could be because these SCD-SC patients were a hospital outpatient population who were the more likely to present with complications. It could also be an indicator that patients with SCD-SC were underrepresented in our registry.

## Conclusion

The SPARCO-Kumasi site has developed capacity (skills and infrastructure) to design, manage, and analyze data in the REDCap SCD registry.

The REDCap registry will provide longitudinal data of the population that would improve the knowledge of SCD in Africa and serve as a resource for further research in SCD. There is also a need to hasten and enhance transition care for the growing adolescent population.

## Limitations

The registry is a hospital-based registry and enrolled only patients who were seeking care at the Kumasi Center for SCD at the Komfo Anokye Teaching Hospital (KCSCD-KATH), a tertiary facility hosting the main sickle cell clinic in the middle and northern belt of Ghana. It therefore excludes patients receiving treatment at other satellite health facilities in the city of Kumasi and other health facilities in Ghana.

## Data Availability

The raw data supporting the conclusions of this article will be made available by the authors, without undue reservation.
