# Supplementary material for: Establishing a Sickle Cell Disease Registry in Africa: Experience From the Sickle Pan-African Research Consortium, Kumasi-Ghana
Source: Front Genet. 2022 Feb 24;13:802355. doi: 10.3389/fgene.2022.802355 (PMC8908904; doi:10.3389/fgene.2022.802355)
Supplement: Supplementary file 1 [file Table1.DOCX]

Table S1: List of database parameters

| **Field Label** | **Choices, Calculations, OR Slider Labels** |
| --- | --- |
| Record ID |  |
| Enrollment pathway | 0, Clinic \| 1, Mass registration \| 2, Home visit |
| Name of person signing consent |  |
| Relationship to participant | 0, Biological Mother \| 1, Biological Father \| 2, Grandparent \| 3, Aunt/Uncle \| 4, Self \| 5, Other |
| Other relation |  |
| Name of Participant/Child |  |
| Consent indication | 0, Thumbprint\|1, Signature |
| Consent Date |  |
| Person Obtaining Consent |  |
| OP Unit Number |  |
| Patient ID/ LHIMS Number |  |
| Visit Date (dd/mm/yyyy) |  |
| Hospital Name | 0, KATH \| 1, KNUST \| 3, Kumasi South |
| Diagnosis Pathway | 0, Non-NSP\|1, NSP |
| NSP Number |  |
| First Name |  |
| Middle Name |  |
| Last Name |  |
| Date of Birth (dd/mm/yyyy) |  |
| Date of Birth Correctness | 1, exact date \| 2, exact day \| 3, month and year \| 4, year only \| 5, unknown |
| Calculated age at recruitment (yr) | rounddown(datediff ([date_of_birth], [consent_date], "y", "dmy"),0) |
| Calculated age at recruitment (mths) | rounddown(datediff ([date_of_birth], [consent_date], "M", "dmy"),0) |
| Age at registration (months)  {Participant less than 5 years} |  |
| Age at registration (completed years)  {Participant more than 5 years} |  |
| Marital Status | 1, Single\|2, Married\|3, Other\|4, Cohabiting\|5, Divorced\|6, Widowed |
| Sex | 1, Male \| 0, Female |
| Occupation |  |
| Religion | 0, None\|1, Christian\|2, Muslim\|3, Traditionalist\|4, Other |
| Other religion |  |
| Educational Status | 0, None \| 1, Basic \| 2, JHS \| 3, SHS/Vocational \| 4, Tertiary |
| Region |  |
| Residence |  |
| Residential Status | 1, Rural \| 2, Semi-urban \| 3, Urban |
| Digital Address System |  |
| Telephone1 |  |
| Telephone2 |  |
| Year of Diagnosis |  |
| SCD Test Result (SS/SBThal/SC) | 1, SS \| 2, SBThal \| 3, SC \| 4, S-beta thal zero (nought) \| 5, S-beta thal plus |
| ABO Blood Group | 1, A+ \| 2, A- \| 3, B+ \| 4, B- \| 5, O+ \| 6, O- \| 7, AB+ \| 8, AB- \| 9, Unknown |
| Pneumococcal Vaccination up to date | 0, No\|1, Yes\|9, Unknown |
| Penicillin V(prophylaxis) | 1, Yes\|0, No\|3, NA\|9, Unknown |
| Folic Acid |  |
| Using Hydroxyurea |  |
| Date of Initiation of Hydroxyurea Therapy |  |
| Anti Malaria Prophylaxis | 1, Yes\|0, No\|3, NA\|9, Unknown |
| Visit date (dd/mm/yyyy) |  |
| Temperature |  |
| Weight (Kg) |  |
| Length/Height (cm) |  |
| Pulse Rate (bpm PULSE) |  |
| Respiratory Rate (cpm) |  |
| Oxygen Saturation (%) |  |
| Systolic Blood Pressure (mmHg SBP) |  |
| Diastolic blood Pressure (mmHg DBP) |  |
| Head Circumference (cm) |  |
| Reason for Visit | 0, Routine\|1, Acute\|2, Review\|3, First visit |
| Admissions | 1, Yes\|0, No |
| Admission details |  |
| Transfusion | 0, No\|1, Yes\|9, Not assessed |
| Units of Blood |  |
| Details of Transfusion |  |
| Nutrition | 0, Normal\|1, Abnormal\|9, Not assessed |
| Details of Nutrition |  |
| Growth | 0, Normal\|1, Abnormal\|9, Not assessed |
| Details of Growth |  |
| Development | 0, Normal\|1, Abnormal\|9, Not assessed |
| Details of Development |  |
| Menstral/Sexual | 0, Normal\|1, Abnormal\|9, Not assessed |
| Details Menstral/Sexual |  |
| Ferile Illness | 0, None\|1, Present\|2, Past\|9, Not assessed |
| Details of Ferile illness |  |
| Pain | 0, None\|1, Present\|2, Past\|9, Not assessed |
| Details of Pain |  |
| Enuresis | 0, None\|1, Present\|2, Past\|9, Not assessed |
| Details of Enuresis |  |
| Priaprism | 0, None\|1, Present\|2, Past\|9, Not assessed |
| Details of Priaprism |  |
| CNS | 0, None\|1, Present\|2, Past\|9, Not assessed |
| Details of CNS |  |
| Cough | 0, None\|1, Present\|2, Past\|9, Not assessed |
| Details of Cough |  |
| Difficult in Breathing | 0, None\|1, Present\|2, Past\|9, Not assessed |
| Details of Difficult in Breathing |  |
| Other Symptoms | 0, None\|1, Present\|2, Past\|9, Not assessed |
| State the details of other symptoms |  |
| Are you Pregnant | 1, Yes\|0, No\|3, NA\|9, Unknown |
| Date of EDD (dd/mm/yyyy) |  |
| Additional Interim History |  |
| Social Family Relations |  |
| School Attendance/Performance |  |
| Immunization Done | 1, DPT/dT\|2, Polio\|3, Hib\|4, Hep\|5, MMR\|6, Pneumo |
| Skin | 0, Normal\|1, Abnormal\|9, Not assessed |
| Abnormal skin details |  |
| Pallor | 1, Yes\|0, No |
| Head | 0, Normal\|1, Abnormal\|9, Not assessed |
| Abnormal head details |  |
| Ears | 0, Normal\|1, Abnormal\|9, Not assessed |
| Abnormal ear details |  |
| Anaemic | 1, Yes\|0, No |
| Details of Anaemia |  |
| Jaundice | 0, Nil\|2, Tinge\|3, Moderate\|4, Deep |
| Details of Jaundice |  |
| Eyes | 0, Normal\|1, Abnormal\|9, Not assessed |
| Other abnormal eye details |  |
| Fundi | 0, Normal\|1, Abnormal\|9, Not assessed |
| Abnormal fundi details |  |
| Nose | 0, Normal\|1, Abnormal\|9, Not assessed |
| Abnormal nose details |  |
| Mouth | 0, Normal\|1, Abnormal\|9, Not assessed |
| Abnormal mouth details |  |
| Teeth | 0, Normal\|1, Abnormal\|9, Not assessed |
| Abnormal teeth details |  |
| Throat | 0, Normal\|1, Abnormal\|9, Not assessed |
| Abnormal throat details |  |
| Neck | 0, Normal\|1, Abnormal\|9, Not assessed |
| Abnormal neck details |  |
| Nodes | 0, Normal\|1, Abnormal\|9, Not assessed |
| Abnormal nodes details |  |
| Chest | 0, Normal\|1, Abnormal\|9, Not assessed |
| Abnormal chest details |  |
| Lungs | 0, Normal\|1, Abnormal\|9, Not assessed |
| Abnormal lungs details |  |
| Heart | 0, Normal\|1, Abnormal\|9, Not assessed |
| Abnormal heart details |  |
| Abdomen | 0, Normal\|1, Abnormal\|9, Not assessed |
| Abnormal abdomen details |  |
| Liver | 0, Normal\|1, Abnormal\|9, Not assessed |
| Abnormal liver details |  |
| Spleen | 0, Normal\|1, Abnormal\|9, Not assessed |
| Spleen Palpable (size) |  |
| Other abnormal spleen details |  |
| Genitalia | 0, Normal\|1, Abnormal\|9, Not assessed |
| Abnormal genitalia details |  |
| Vertebrae | 0, Normal\|1, Abnormal\|9, Not assessed |
| Abnormal vertebrae details |  |
| Extremities | 0, Normal\|1, Abnormal\|9, Not assessed |
| Abnormal extremities details |  |
| Joints | 0, Normal\|1, Abnormal\|9, Not assessed |
| Abnormal joints details |  |
| Neurological | 0, Normal\|1, Abnormal\|9, Not assessed |
| Abnormal Neurological details |  |
| Lab test results | 0, None\|1, FBC/CBC\|2, Biochemistry\|3, LFT\|4, HB Electrophoresis\|5, Urine RE\|6, Blood Culture\|7, Other\|8, ESR\|9, RFT\|10, MPs |
| FBC test date |  |
| WBC (10^3uL) |  |
| RBC (10^6uL) |  |
| HB (g/dL) |  |
| MCV (fL) |  |
| MCH (pg) |  |
| MCHC (g/dl) |  |
| PLT (10^3ul) |  |
| RETICS % |  |
| ESR |  |
| Mps | 10, RDT Negative\|11, RDT Positive\|20, Microscopy negative\|21, Microscopy Positive |
| LFT Date |  |
| AST (u/l) |  |
| ALT (u/L) |  |
| ALP (u/L) |  |
| GGT (u/L) |  |
| Albumin (g/L) |  |
| Bilirubin total (umol/L) |  |
| Total protein (g/L) |  |
| Globulin (g/L) |  |
| Serum Electrolyte date |  |
| Sodium (Na) mmol/L |  |
| Potassium (K) mmol/L |  |
| Chloride (Cl) mmol/L |  |
| Total CO2 mmol/L |  |
| RFT Date |  |
| Urea (mmol/L) |  |
| Creatinine (mmol/L) |  |
| BUN to Creatinine Ratio |  |
| Other tests results |  |
| HB Electrophoresis date |  |
| Hb Genotype |  |
| Appearance |  |
| Colour |  |
| pH |  |
| Protein | 0, Negative\|1, Positive |
| Glucose | 0, Negative\|1, Positive |
| Ketone | 0, Negative\|1, Positive |
| Blood | 0, Negative\|1, Positive |
| Bilirubin | 0, Negative\|1, Positive |
| Nitrite | 0, Negative\|1, Positive |
| Urobilinogen | 0, Normal\|1, Abnormal |
| Specific gravity |  |
| Pus cells (hpf) |  |
| Red blood cells (/hpf) |  |
| Epithelial Cells (/hpf) |  |
| RBCs | 0, Not seen\|1, Present |
| Crystals | 0, Not seen\|1, Present |
| Casts | 0, Not seen\|1, Present |
| Yeast cells | 0, Not seen\|1, Present |
| T. Vaginals | 0, Not seen\|1, Present |
| S. Haematobium | 0, Not seen\|1, Present |
| Spermatozoa | 0, Not seen\|1, Present |
| Clue Cells | 0, Not seen\|1, Present |
| Others | 0, Not seen\|1, Present |
| Other Urine RE findings |  |
| Imaging Results Available | 0, None\|1, X-ray\|2, TCD\|3, Ultrasound\|4, CT\|5, MRI |
| X-ray Date |  |
| Xray results |  |
| TCD date |  |
| TCD result MCA_left |  |
| TCD result MCA_Right |  |
| TCD result ICA_left |  |
| TCD result ICA_Right |  |
| TCD remarks |  |
| Ultrasound date |  |
| Ultrasound results |  |
| CT Date |  |
| CT results |  |
| MRI Date |  |
| MRI results |  |
| Overall assessment |  |
| Issues and Problems |  |
| Tests ordered | 1, FBC \| 2, Electrolyte \| 3, X-ray \| 4, Ultrasound \| 5, TCD \| 6, LFT \| 7, RFT \| 8, Retics \| 9, MPs \| 10, BUE Cr \| 11, Blood Culture \| 12, Urine RE |
| Routine Medications | 1, Penicilin V 125mg bd\|2, Pennicillin V 250mg bg\|3, Folic acid 5mg dly\|4, Folic Acid 10mg dly\|5, HU\|6, Haematinics\|7, Analgesics |
| Medications Prescribed |  |
| Other recommendations for issues and problems |  |
| Referrals |  |
| Next Routine Visit (dd/mm/yyyy) |  |
| Next Visit Correctness | 1, exact day, month and year \| 2, date unknown |
| Attending Doctor |  |
| Today's Education Topics |  |
